# Quantitative Prediction of Residual Stress, Surface Hardness, and Case Depth in Medium Carbon Steel Plate Based on Multifunctional Magnetic Testing Techniques

**DOI:** 10.3390/s25092812

**Published:** 2025-04-29

**Authors:** Changjie Xu, Xianxian Wang, Haijiang Dong, Juanjuan Li, Liting Wang, Xiucheng Liu, Cunfu He

**Affiliations:** 1 School of Emergency Equipment, North China Institute of Science and Technology, Langfang 065201, China; lantianljj@163.com; 2 College of Mechanical & Energy Engineering, Beijing University of Technology, Beijing 100124, China; 3 Mechanical and Electrical Engineering Institute, Beijing Polytechnic University, Beijing 100176, China; wangxianxian@bpi.edu.cn; 4 China United Test & Certification Co., Ltd., Beijing 101407, China; dhaijiang2008@163.com; 5 School of Mechanical Engineering, Inner Mongolia University of Science and Technology, Baotou 014010, China; litingwang2021@imust.edu.cn; 6 School of Information Science and Technology, Beijing University of Technology, Beijing 100124, China; xiuchliu@bjut.edu.cn (X.L.); hecunfu@bjut.edu.cn (C.H.)

**Keywords:** quantitative prediction, multifunctional magnetic testing, residual stress, surface hardness, case depth, MLR inversion

## Abstract

In this study, the methods of tangential magnetic field (TMF), magnetic Barkhausen noise (MBN), and incremental permeability (IP) were employed for in the simultaneous, quantitative prediction of target properties (bidirectional residual stress, surface hardness, and case depth) in the 45 steel plate. The bidirectional magnetic signals and target properties were measured experimentally. The results of Pearson correlation analyses revealed that most parameters of the MBN and IP signals are strongly correlated with both residual stress and surface hardness under the influence of multiple target properties. The multiple linear regression (MLR) model demonstrated highly accurate quantitative prediction of residual stress and hardness in the y-direction. However, the simultaneous prediction of residual stress and case depth in the x-direction proved less effective than expected. To address this limitation, an inversion method was developed based on the regression model with the single parameter as the dependent variable and the target properties as the independent variable. By incorporating known magnetic parameters and target properties, the model effectively determined the unknown target properties. After applying the method, the coefficient of determination (R^2^) for x-direction residual stress increased from 0.89 to 0.96 and the absolute error (AE) of case depth decreased from 0.10 mm to 0.04 mm for case depths below 0.15.

## 1. Introduction

Laser hardening is an effective method for the surface strengthening of medium carbon steels. After hardening, measurements of residual stress, hardness, and case depth are essential not only to optimize the process parameters, but also to enhance the product quality and performance. Conventional methods that can only measure the single target property are either destructive or expensive. To overcome this technological bottleneck, researchers at Fraunhofer-IZFP [1,2] have developed a multifunctional micromagnetic technique. The technique works by simultaneously acquiring multiple magnetic signals, including magnetic Barkhausen noise (MBN), tangential magnetic field (TMF), incremental permeability (IP), hysteresis loop (HL), and eddy current (EC) signals. Feature parameters are extracted to calibrate the material properties, enabling the non-destructive quantitative assessment of residual stresses and mechanical properties (e.g., surface hardness) [3,4]. When a ferromagnetic material is subjected to an external magnetic field, both microstructural features and stress states influence the structure and dynamic properties of magnetic domains [5,6,7]. Domain wall motion can be impeded by stress-induced pinning effects [8,9]. The microstructure determines the magnetic domain structure and motion properties [10]. Thus, the characterization of magnetic domains with reversible and irreversible motion corresponds to a variety of material properties such as microstructure, residual stress, mechanical properties, and hardness.

Studies have shown that sometimes, single magnetic parameters can directly reflect the trends of material mechanical properties. For example, MBN parameters demonstrate a good linear relationship with the stress and hardness of hardened specimens [11,12] and decarburised specimens [13]. The trends of some of its parameters are highly consistent with the distribution pattern of residual stresses in weldments [14]. Similarly, some parameters of the tangential magnetic field (TMF) not only have a linear correlation with residual stresses [15], but also show a monotonic variation with the depth of the hardened layer [16,17]. Parameters from incremental permeability, hysteresis loops, and eddy current signals also show linear correlations with mechanical properties [18,19,20]. However, despite the fact that these single magnetic parameters reveal certain linear patterns with the target properties, their predictive accuracy is still limited (the single-parameter goodness-of-fit of existing studies is generally less than 0.9), making it difficult to meet the need for high precision assessment. This limitation indicates that it is difficult to comprehensively and accurately characterize the overall performance of materials by relying on a single magnetic parameter. Furthermore, micromagnetic parameters and mechanical properties often exhibit complex nonlinear relationships [21,22,23]. Consequently, the further development of multi-parameter fusion analysis methods is essential to enhance the assessment.

When using micromagnetic methods for the quantitative prediction of mechanical properties, the key issue lies in establishing stable and highly accurate models using calibrated experimental data. Multiple linear regression models are commonly used because of their simplicity and stability. Recently, machine learning (e.g., neural networks, support vector regression, random forest, and back-propagation), with its powerful ability of feature mining and data fusion, has been increasingly utilized for the efficient characterization of stress and mechanical properties in material through the hybridization of different techniques [24,25,26]. Feature screening combined with MLR methods has been jointly employed to predict the surface hardness and case depth of hardened 45 steel parts [27].

Previous research by the authors demonstrated that neural networks have advantages in prediction accuracy over multiple linear regression models. However, when the magnetic signals are influenced by multiple factors and the case depth varies over a relatively narrow range, the neural network model may suffer from overfitting problems. In this study, micromagnetic measurements were integrated with MLR for the quantitative prediction of three target properties (residual stress, surface hardness, and case depth) of hardened medium carbon steel parts. Pearson correlation coefficients between the magnetic parameters and the target properties were analyzed to reveal correlation patterns between the feature parameters of different types of magnetic signals and the target properties when they are affected by multiple target properties. The multiple linear regression models were established to quantitatively predict the target parameters. Finally, the prediction results were optimized using a single-parameter inversion method. Previous studies have shown that machine learning modeling techniques, such as neural networks, are highly accurate in predicting material properties, and that the prediction performance improves with larger datasets. However, they are time-consuming to train and risk overfitting. In contrast, multivariate linear regression models are accurate, stable, and computationally fast when numerous magnetic parameters exhibit strong linear correlations with material properties, making them suitable for rapid response or small datasets. Nevertheless, the magnetic signals were influenced by the combination of factors, and the varying influence weights of the target properties led to variations in the correlation, which affected the prediction accuracy of some target properties.

This study analyzed Pearson correlation coefficients between the magnetic parameters and the target properties (residual stress, surface hardness, and case depth). The magnetic parameters with a strong linear correlation were screened out and multiple linear regression models were constructed to quantitatively evaluate the target properties. To address the limitations, the single-parameter inversion method was proposed, where the regression models for individual magnetic parameters were built, combined with the known magnetic parameters with the target properties data, to inversely determine unknown target properties to improve the prediction accuracy.

The remainder of this paper is organized as follows. Section 2 details the experimental methodology, including sample preparation, multifunctional magnetic testing procedures, and measurements of the target properties (bidirectional residual stress, surface hardness, and case depth). Section 3 provides a comprehensive correlation analysis that examines the relationships between various magnetic parameters and target properties, focusing on individual and combined property effects. Section 4 evaluates the predictive accuracy of the multiple linear regression (MLR) model for target property estimation and assesses the effectiveness of single-parameter inversion methods for determining x-direction residual stress and case depth. Finally, Section 5 summarizes the key findings and conclusions.

## 2. Experiment

### 2.1. Specimen Preparation

The specimen is made of high-quality carbon structural steel 45. The chemical composition is given in Table 1. The 45 steel has excellent strength and machinability. Its basic mechanical properties are a Young’s modulus of about 210 GPa, yield strength ≥ 350 MPa, and tensile strength ≥ 600 MPa. Laser hardening can significantly improve the surface hardness and mechanical properties (yield strength ≥ 1000 MPa) of the material, but creates residual stresses.

**Figure 1:**
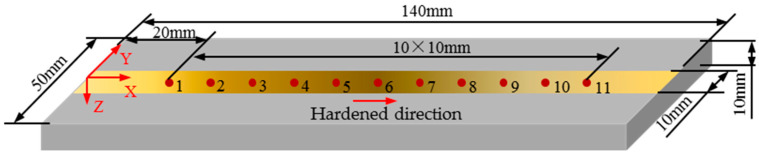
Schematic diagram of induction-hardened specimen and test points.

The specimen size was machined from a steel plate with dimensions of 140 mm × 50 mm × 10 mm. A coordinate system was established, as shown in Figure 1, with the origin set at the midpoint of the left edge on the top surface of the steel plate. The position 20 mm to the right of the origin was marked as test point No. 1, and subsequent test points were marked every 10 mm along the direction of the *x*-axis and its extension line until a total of 11 test points were marked. A CO^2^ laser with an output power of 2000 W, a set scan rate of 400 mm/min, and a spot size of 10 mm^2^ were used to perform laser hardening along the *x*-axis from the origin of the coordinates. In order to control the specific values of the residual stress, surface hardness, and case depth of the hardened strip, the laser height was adjusted.

Bidirectional residual stress measurements were conducted via X-ray diffraction along the x-direction (parallel to the laser-hardened zone) and y-direction (perpendicular to the hardened zone) at all 11 test points using a Pulstec U360 X-ray (Hamamatsu, Japan) stress analyzer. The measurement results are detailed in Table 2. The surface hardness (Table 2) at these test points was measured using a Wilson hardness tester with an indenter load of 1.96 N (equivalent to 0.2 kgf).

**Figure 2:**
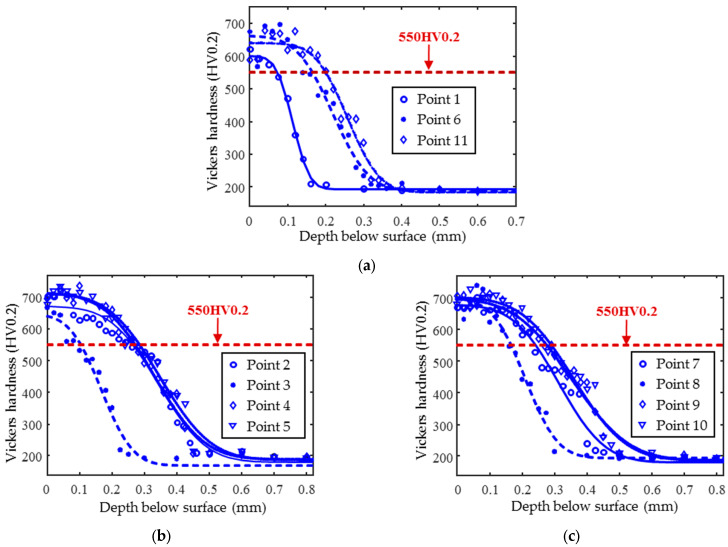
Hardness test results and the fitted curves of hardness–depth profiles for 11 points of the induction-hardened specimen (**a**) 1, 6, and 11 points; (**b**) 2, 3, 4, and 5 points; and (**c**) 7, 8, 9, and 10 points.

The hardened specimen can be divided into three distinct zones: the hardened layer, the transition layer, and the matrix, from the surface to the core. To quantitatively estimate the effective case depth, the hardness values are measured continuously along each cross-section from the surface to the core zone. The following sampling principles should be followed: a high density spacing of 20–25 μm is used in the near-surface region (where the hardness gradient is most pronounced) and in the transition zone, while a coarser spacing of 100 μm is used in the substrate region to ensure sufficient data density in critical areas. The total number of measurement points ranges from 11 to 28, with a minimum of 8 points in the surface and transition zones and 3–5 points in the substrate region, to ensure that the complementary error function (ERFC) accurately captures the hardness variations. Nonlinear least squares fitting is used, requiring good fit (R^2^ > 0.96) and uniformly distributed residuals. If abrupt hardness gradients are observed, localized refinement to 10 μm spacing is recommended to improve the fitting accuracy. The hardness profiles of the 11 points were fitted using the complementary error function method, as shown in Figure 2. According to the national standards, when the hardness value drops to 550HV0.2, the depth of the corresponding transverse coordinate from the surface is defined as the effective case depth (subsequently referred to as case depth). The measurement results of case depth are detailed in Table 2. The measured case depth at 11 locations ranges from 0.07 to 0.29 mm, showing an initial increase (0.07–0.28 mm) followed by minor fluctuations. Peaks occur at points 5, 9, and 10 (0.28–0.29 mm), indicating local heterogeneity, while 72.7% of the values cluster in the 0.20–0.29 mm range, demonstrating reasonable consistency in case depth thickness.

**Figure 3:**
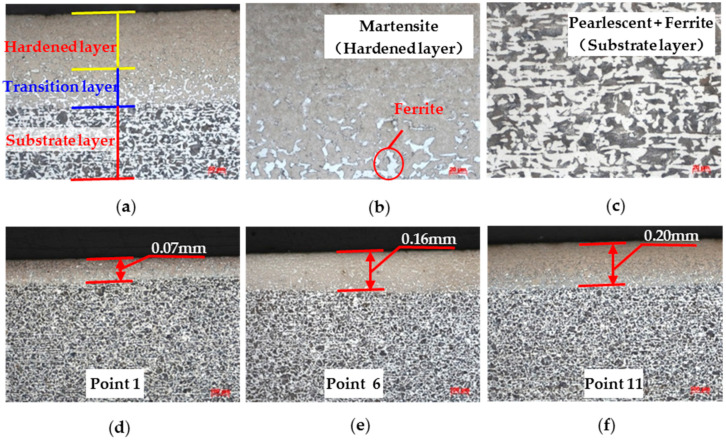
Optical micrographs of the microstructures in hardened specimen: (**a**) different layers; (**b**) hardened layer; (**c**) substrate layer; and case depth with point (**d**) 1; (**e**) 6; and (**f**) 11.

To examine the metallographic microstructure of the hardened steel plate at different locations, samples were taken from 1, 6, and 11 test points on the specimen. The metallographic specimens were prepared through sequential polishing, etching with nitric alcohol, and washing and drying with ethanol. The results of the metallographic microscopic observation are shown in Figure 3. After the hardening treatment, a hardened layer was formed on the surface of the specimen and its material structure was transformed into hard martensite, as shown in Figure 3b. There was a transition layer between the hardened layer and the substrate, in which the martensite was permeated with ferrite. As evident in Figure 3c, the matrix material primarily consists of pearlite and ferrite. The micrographs clearly reveal that the case depth at test points No. 1, No. 6, and No. 11 exhibited a progressive increase. Moreover, the value of case depth estimated via metallographic analysis coincides with the estimate previously derived using the complementary error function method.

### 2.2. Multifunctional Magnetic Testing

**Figure 4:**
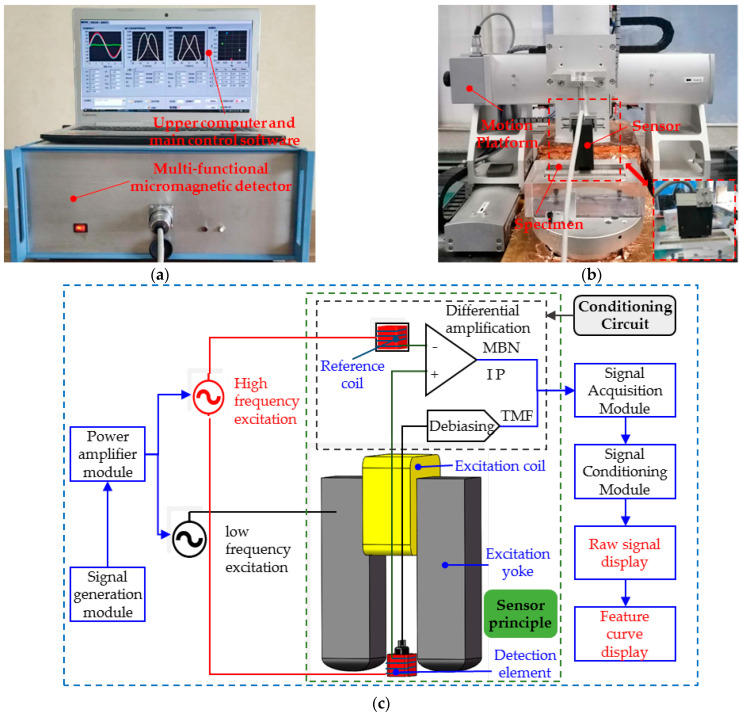
Experimental system. (**a**) Micromagnetic detector; (**b**) motion platform with sensor; (**c**) and block diagram of micro-magnetic detector.

A multifunctional micro-magnetic detector (shown in Figure 4a) developed by the Research Center for NDT&E in Beijing University of Technology was employed for the experiments. The detector consists of the master device, the upper computer with the main control software, and a micromagnetic sensor. The sensor consists of a U-shaped yoke wound with an excitation coil, dual detection elements (the Hall element and the twisted pair induction coils), and a signal conditioning circuit (shown in Figure 4c). Hall elements and induction coils are configured on the inside of the U-shaped magnetic core, which is embedded in the preloaded spring assembly to maintain the consistency of the lifting distance between the detection element and the specimen surface. The specimen is magnetized by the intermittent superimposed excitation of high and low frequencies to achieve multi-functional micro-magnetic signal testing. The low-frequency magnetic field was generated by a U-shaped electromagnet made by winding enameled copper wire (approximately 0.35 mm diameter, 350 turns) around the top beam of a U-shaped yoke made of laminated silicon steel. The high frequency magnetic field was produced by a rectangular coil made of twisted wire. The twisted induction coil has an air core with about 200 turns of enameled copper wire with a diameter of 0.05 mm, a coil height of about 15 mm, and external dimensions of about 6 mm × 4 mm. Two coils are made up of a twisted pair of wires, one of which is a detection coil placed in the middle of the electromagnet, near the surface of the specimen. It is used to detect magnetic Barkhausen noise signals or incremental permeability signals. The other acts as a high-frequency reference coil. The direction of the high-frequency magnetic field is perpendicular to the direction of the low-frequency magnetic field. The detector is capable of testing three types of micromagnetic signals simultaneously: tangential magnetic field strength (TMF), Barkhausen magnetic noise (MBN), and incremental permeability (IP), enabling the quantitative prediction of bi-directional stress and hardness, as well as case depth via calibration.

The block diagram in Figure 4 illustrates the signaling flow of the micromagnetic detector. The signal generator module excited the superimposed low-frequency and high-low-frequency excitation signals at different times, and the power amplifier module amplified them. When only low-frequency excitation was applied (sinusoidal signal with an amplitude of 2 V and a frequency of 200 Hz), the sensor excitation coil (wrapped around the top of the excitation yoke) generated an alternating magnetic field to locally magnetize the specimen. The Hall element (S39ET, dynamic operating range + 1000 Gs, sensitivity 1.4 mV/Gs) collected the TMF signal and the detection coil acquired the MBN signal synchronously. And then the conditioning circuit of the sensor corrected the TMF signals via de-biasing, while the MBN signals were amplified. During the superimposed excitation of high frequencies (sinusoidal signal with an amplitude of 2 V and frequency of 100 kHz) and low frequencies (sinusoidal signal with an amplitude of 2 V and frequency of 200 Hz), the twisted-pair induction coils are energized with a high-frequency excitation signal and the detection signal is picked up at the same time. To remove the effect of mutual inductance, the reference coil (configured in the conditioning circuit above the top beam of the U-shaped yoke) signal and the detection signal are differentially amplified to obtain the IP signal. After the signal is acquired by the signal acquisition module, the conditioning module of the master device filters the TMF and MBN signals, demodulates the IP signal, and then outputs them to the upper computer software for display.

For detection, a four-axis moving platform (Figure 4b) was used to precisely locate the sensor to reduce uncertainty. The NI PXIe-8880 host computer, NI PXI-7340 controller card, and NI PXIe-1071 chassis (Austin, TX, USA), combined with the LabVIEW software (version 2024 Q1) and control stepper motors were used to move the sensors along the defined path. The sensor axis is always coincident with the normal detection point during detection. The sensor first detected points 1–11 along the *x*-axis, then returned to the starting point and repeated the operation until the detection was complete.

**Figure 5:**
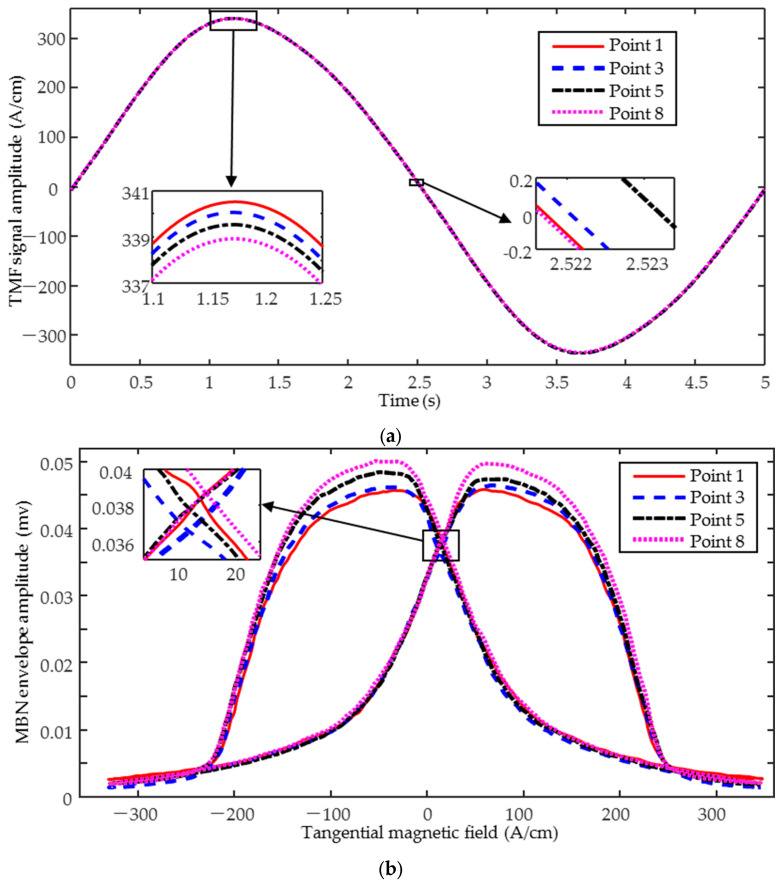

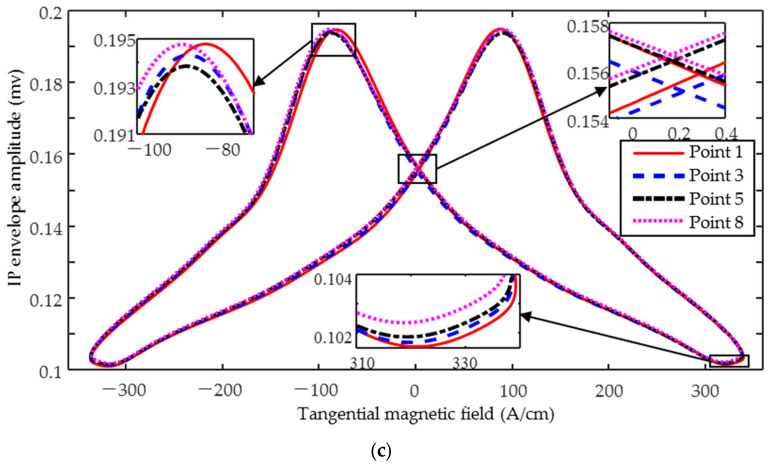
Typical magnetic signals (**a**) tangential magnetic field; (**b**) MBN butterfly curve; and (**c**) IP butterfly curve.

For bidirectional stress prediction and the bidirectional magnetic signals were tested at eleven preset positions, with the sensors excited parallel (along the *x*-axis) and perpendicular (along the *y*-axis) to the hardening direction, respectively. To improve the accuracy of the quantitative prediction, each position was detected three times, five magnetization cycle signals were collected each time, and the middle three stable magnetization cycles were selected for subsequent feature extraction. The MBN butterfly curve (Figure 5b) is plotted with the TMF fundamental amplitude as the horizontal coordinate and the amplitude of the MBN envelope curve after sliding averaging as the vertical coordinate. The IP butterfly curve (Figure 5b) is obtained by using the amplitude of the demodulated IP real part as the vertical coordinate.

Harmonic analysis is performed on the TMF signal to extract parameters such as fundamental and odd harmonic amplitude and phase. Parameters such as the peak value of the MBN and IP single magnetization cycle and the half-peak width of the butterfly curve are calculated, and a total of 25 magnetic feature parameters are extracted, as shown in Table 3.

**Figure 6:**
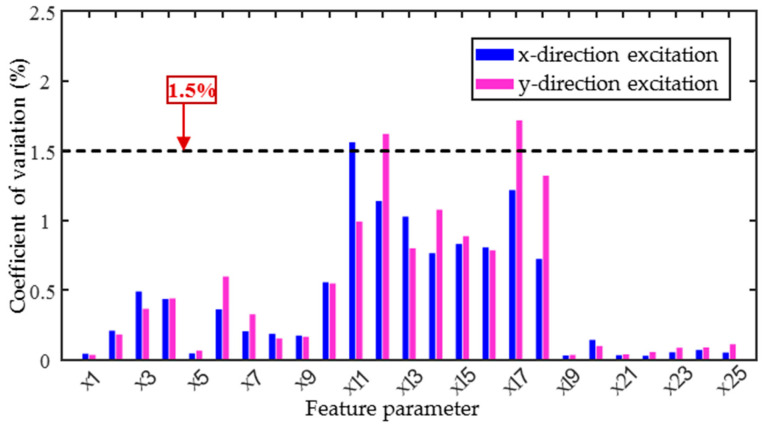
Coefficients of variation in each feature parameter of specimen.

From the three sets of collected data, the coefficient of variation δ (δ = σ/μ, where σ is the standard deviation and μ is the mean value) was calculated for each micromagnetic parameter. Larger coefficients of variation indicate greater differences in the repeated measurement data, which may affect the accuracy of the subsequent quantitative prediction model. Figure 6 shows the results of the coefficient of variation calculation for the 25 magnetic parameters. From the figure, it can be seen that the coefficients of variation for the IP feature parameters are all less than 0.5%, indicating the high degree of stability of the repeated test data. Similarly, the coefficients of variation for both the TMF and MBN feature parameters are also less than 2%, confirming that the differences in the repeated test data for these parameters are relatively small. Therefore, based on the assessment of data stability, all parameters are suitable for the subsequent prediction models.

## 3. Correlation Analysis

Residual stress, surface hardness, and case depth are the target properties for quantitative prediction using magnetic feature parameters. The Pearson correlation coefficient was calculated to evaluate the dependency of magnetic parameters on target properties. Magnetic parameters with a strong correlation with the target properties were used in subsequent multiple linear regression modeling to improve the accuracy of the predictions.

**Figure 7:**
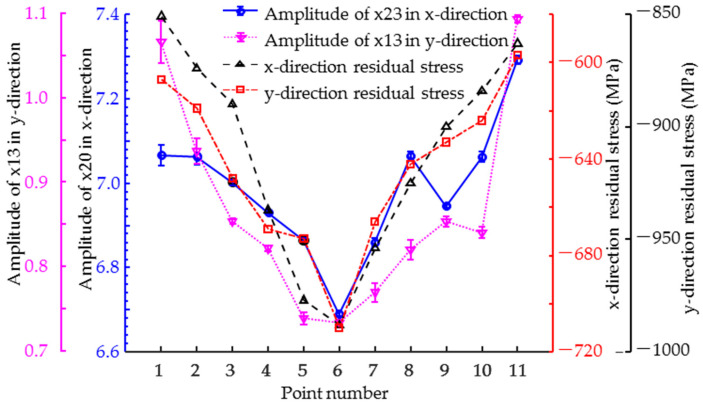
Measured profiles of typical feature parameters and residual stress in all tested points.

When the residual stresses coincide with the direction of excitation, they have the most significant effect on the magnetic signal. Therefore, it is necessary to focus only on the magnetic feature parameters with excitation and residual stress in the same direction and to calculate the Pearson correlation coefficients between these parameters and the residual stress. As shown in Figure 7, the variation curves of the typical feature parameters x13 and x23 with the position of the test point have the same trend as the distribution curve of the residual stress in the corresponding direction. This figure tentatively shows that x13 and x23 exhibit a positive correlation with residual stress.

**Figure 8:**
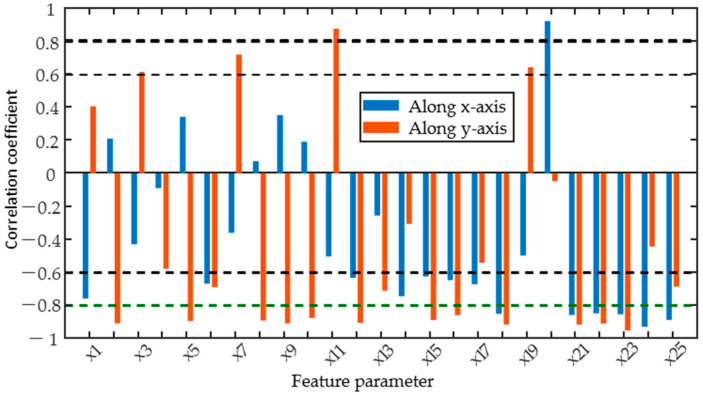
Correlation coefficient between feature parameters and residual stress (stress and excitation in the same direction).

To quantitatively assess the sensitivity of individual magnetic parameters to residual stresses, the correlation coefficients between the candidate parameters and residual stresses were calculated and the results are shown in Figure 8. The positive and negative Pearson correlation coefficients for most magnetic parameters depend on the measurement direction. When the direction of magnetization was changed from along the *x*-axis to the *y*-axis, the sign of the correlation coefficients between the magnetic feature parameters x1, x3, x7, and x11 of the TMF and the residual stress varied from negative to positive values. However, the MBN and IP signals are more significantly affected by residual stress, as indicated by the fact that the correlation coefficients between most of the magnetic feature parameters extracted from these signals and residual stress exceed 0.6, showing a strong correlation. Furthermore, when both the excitation direction and the residual stress are along the *y*-axis, the correlation coefficients of most of the magnetic parameters are greater than 0.8, indicating a high correlation with the residual stress, while the correlation coefficient of x20–x25 of the IP signal exceeds 0.8 when both directions are along the *x*-axis. The reason for this is that the micromagnetic signal in the *y*-axis direction is only affected by residual stress, whereas the combination of residual stress, hardness, and case depth affects the micromagnetic signal in the *x*-axis direction. The change in sign of the Pearson correlation coefficient shows the influence of the direction of the micro-magnetism measurements, suggesting that the direction of excitation must be fully taken into account for the quantitative prediction of residual stresses in laser-hardened specimens using the TMF and MBN methods.

**Figure 9:**
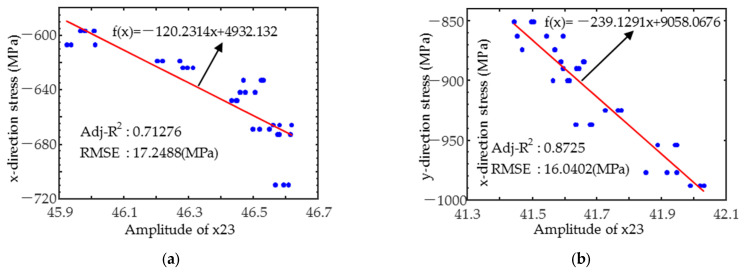

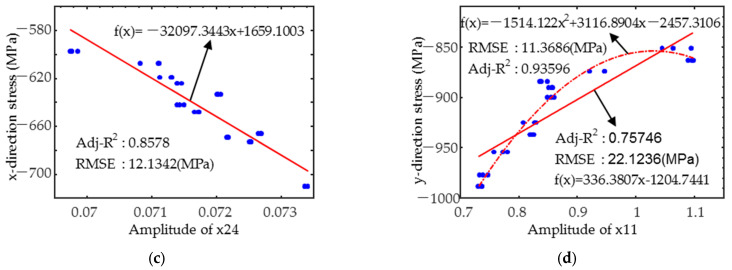
Variation in typical feature parameters (**a**) x23 and x-directional; (**b**) x23 and y-directional; (**c**) x24 and x-directional; and (**d**) x11 and y-directional residual stress.

The linear or quadratic residual stress prediction models (shown in Figure 9) were built by selecting the magnetic parameters that correlated strongly with the residual stresses based on Figure 8. As demonstrated in Figure 9a,b, the magnetic parameter x24 has a good linear relationship with the corresponding residual stress in both directions. Influenced by hardness and case depth, the x-direction had a slightly lower coefficient of determination (R^2^), close to 0.7, while the y-direction was only influenced by the residual stresses, which had a wide range of variation, and the R^2^ was close to 0.9. The root mean square error (RMSE) of both was less than 20 MPa. In the x-direction, the magnetic parameter x24 had the best linear fit to the residual stress (Figure 9c), with an R^2^ greater than 0.85 and an RMSE of less than 13 MPa. As shown in Figure 9d, the linear fit of the parameter x11 to the residual stress in the y-direction is also high, with the R^2^ exceeding 0.75. However, it should be noted that the variation law of x11 with residual stress in the y-direction is more consistent with the quadratic relationship model, which achieves an R^2^ of more than 0.93 and an RMSE of less than 12 MPa. The above examples reveal that the magnetic feature coefficients do not always have a simple linear correlation with the target properties. In view of this, the Pearson correlation coefficient is of greater reference value as a feature screening method when multiple linear regression models are used for the quantitative prediction of target properties.

When the excitation direction is along the *x*-axis, the hardened region lies exactly within the magnetization center region and the magnetic signal accurately reflects the hardness and case depth, so it is only necessary to analyze the relationship between the magnetic parameter and these two factors under the x-direction.

**Figure 10:**
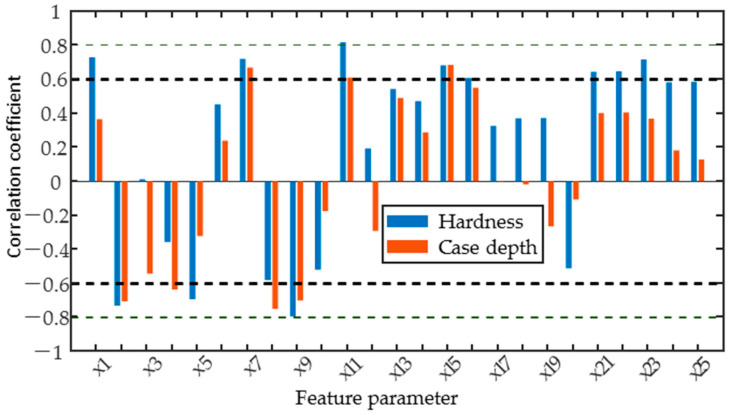
The correlation coefficient between feature parameters and surface hardness.

As shown in Figure 10, by calculating the Pearson correlation coefficients of individual magnetic parameters with hardness and case depth, the results show that the correlation coefficients of most magnetic parameters with hardness are significantly higher than with case depth. The comparison of Figure 8 and Figure 10 shows a variety of correlations between the magnetic parameters and the target properties. There is a strong correlation only with residual stress for some of the magnetic parameters, such as x20. However, more magnetic parameters, such as x1, x21 to x25, are strongly correlated with both residual stress and hardness, while x2, x7 to x9, x11, x15, and x16 are correlated with hardness and case depth. Comprehensive analyses show that the IP signal is sensitive to residual stress and hardness; the MBN signal is influenced by residual stress, hardness, and case depth; and the TMF signal is mainly affected by hardness and case depth.

**Figure 11:**
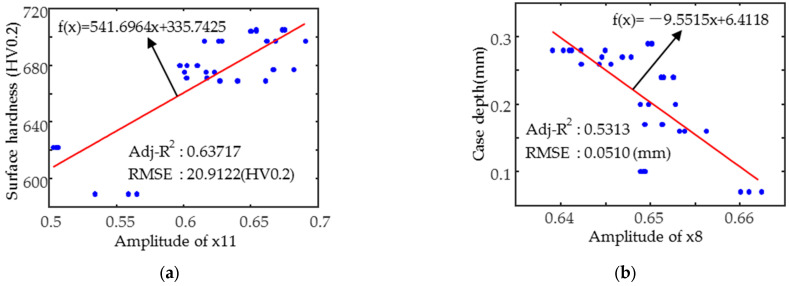
Variation in typical feature parameters (**a**) x11 and surface hardness and (**b**) x8 and case depth.

The magnetic parameter x11, which has the strongest correlation with hardness, was chosen to predict the surface hardness (see Figure 11a), and the results show a poorly fitted straight line with an R^2^ value below 0.65. This is due to the effect of variations in the case depth and the poor homogeneity of the data (the hardness data are concentrated in the 600–720 HV0.2 interval). When predicting the case depth, the best fitting single parameter was x8 (see Figure 11b), which was more poorly fitted, mainly due to the influence of hardness and the small variation in the case depth. However, it is worth noting that the root mean square error (RMSE) of the model is less than 0.06 mm, which is still a reasonable benchmark for the evaluation of the case depth uniformity, and is particularly suitable for rapid assessment or preliminary screening.

## 4. Results and Discussion

### 4.1. Multiple Linear Regression for Quantitative Prediction

Based on the correlation coefficient analyses mentioned above, it can be seen that a number of magnetic parameters have strong linear correlations with residual stress, surface hardness, and case depth. Therefore, the micromagnetic parameters with significant correlations (correlation coefficients greater than 0.6) with these target properties were selected as independent variables, with bidirectional residual stress, surface hardness, and case depth as dependent variables, and then multiple linear regression models were constructed.

From the three sets of repeated experimental data, two sets were randomly selected for model training, while the remaining set of data was used for external validation. A stepwise regression algorithm was applied to train the model, and the values of the micromagnetic parameters and their coefficients used in the final model are shown in Table 4.

Since the y-direction magnetic signals are mainly influenced by the y-direction stress, the yRs (y-direction stress) regression model contains multiple parameters for all three magnetic signals. In contrast, the x-direction magnetic signal is influenced by the combination of x-direction residual stress, surface hardness, and case depth. The xRs (x-direction stress) regression model includes the MBN parameters, the SH (surface hardness) model includes the TMF and IP magnetic parameters, and the ECD (case depth) model includes only a small number of TMF and MBN parameters. The results show that when only a single target property (e.g., residual stress) is involved, all magnetic signals are sensitive to the variation in target property; however, when multiple target properties are involved, the magnetic signals have different sensitivities to the target property, i.e., the weights of the target properties on the magnetic signals are different.

**Figure 12:**
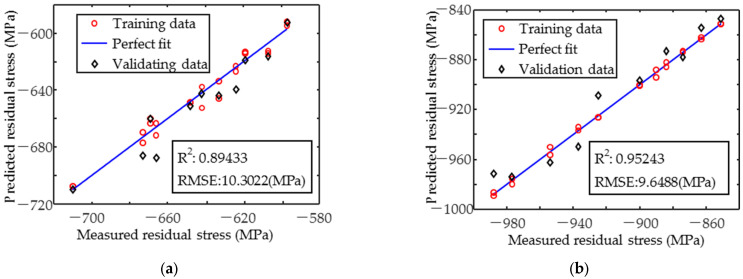
Prediction result of (**a**) x-directional and (**b**) y-directional residual stress.

The results of the residual stress prediction using the multiple linear regression model are shown in Figure 12. The magnetic signal in the y-direction, which is influenced by a single residual stress, is better predicted than in the x-direction, which is influenced by several target properties. In the y-direction, the coefficient of determination R^2^ is greater than 0.95, whereas in the x-direction, the R^2^ is less than 0.9. The RMSE of the x-direction and y-direction is 10.3022 MPa and 9.6488 MPa, respectively.

**Figure 13:**
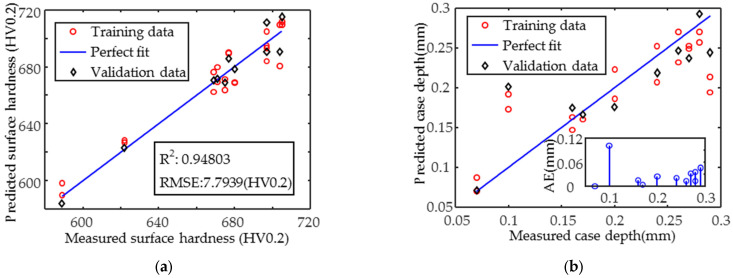
Simultaneous prediction results of (**a**) surface hardness and (**b**) effective case depth.

The accuracy of the hardness prediction model shown in Figure 13a is high, and increasing the amount and improving the homogeneity of the data can further improve the prediction accuracy. In contrast, the case depth prediction result (Figure 13b) is unsatisfactory, with the coefficient of determination R^2^ of 0.72, as the case depth is not a dominant factor affecting the magnetic signal. The absolute error in the inset shows that the data points with an effective case depth of 0.1 mm have a large error of more than 0.1 mm, while the remaining points have an error of less than 0.05 mm. The RMSE is 0.04 mm, and although this value is not yet sufficient for the direct and accurate prediction of the case depth, it can still be used as a valuable reference for assessing the degree of case depth uniformity.

### 4.2. Single Magnetic Parameter Inverse Residual Stress and Case Depth

Based on the above MLR prediction results, the MLR model developed using multiple magnetic parameters had a high prediction accuracy when the magnetic signal was dominated by only a single factor of y-direction residual stress. However, when the magnetic signal is influenced by three target properties (case depth, surface hardness, and residual stress in the x-direction), the predictive performance of the model is unsatisfactory, especially in the prediction of stress in the x-direction and case depth. Considering that the variation in the magnetic feature parameters is the result of the combined effect of several target characteristics, the corresponding individual magnetic parameter regression models are established for the target properties. The known magnetic parameter and target properties information were applied to the model to invert the unknown target property.

A regression model was constructed using a single magnetic parameter as the dependent variable and a target property as the independent variable, combined with the results of a correlation analysis. The aim of this model is to evaluate the degree of influence of each target property on the magnetic parameters and to invert the unknown target property. For modeling, two measurements were used as training data and one as calibration data and the fit was assessed by the coefficient of determination R^2^. As there is a complex mapping relationship between the target properties and the magnetic parameters, not only a simple linear relationship, the quadratic, multiple phase, and exponential terms are added to the model. The results, where both the linear and quadratic terms were included and the R^2^ was greater than 0.7, are presented in Table 4. With the addition of the cubic term to the model, only the R^2^ of the magnetic parameter x24 is improved, as shown in Table 5.

**Figure 14:**
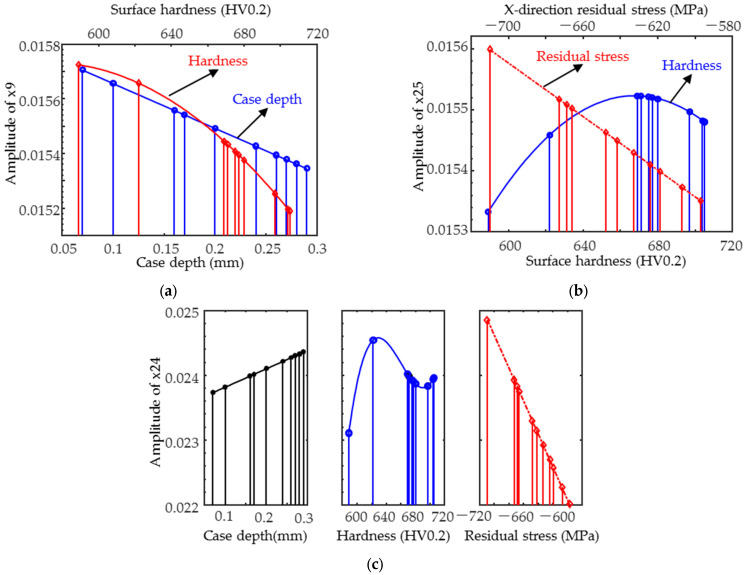
Typical magnetic parameter (**a**) x9; (**b**) x25; and (**c**) x24 variation curves with target properties.

Taking into account the range of variation in each target property, a rough comparison of the weights of each target property on the magnetic parameters in Table 5 has been performed via the method of comparison of normalized coefficients and plotting scatter plots. The calculation of the standardized coefficients of the parameter x2 equation yielded a larger contribution of hardness to the variation in the parameters than that of the case. As in Figure 14a, both hardness and case depth influenced x9, with hardness weighted slightly more. Therefore, the TMF magnetic parameters were only affected by the hardness and hardened layers and were more sensitive to the microstructure, whereas the IP parameters x21–x24 were influenced by the combination of hardness and residual stress with a small weight difference, and the effect of the case was almost negligible. As in Figure 14b, the magnetic parameter x25 varies more rapidly with residual stress, i.e., the residual stress is weighted more.

**Figure 15:**
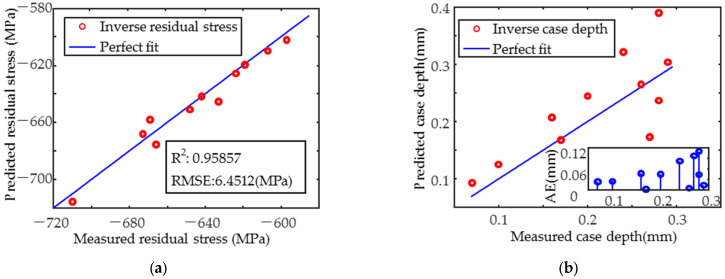
x24 of cubic multiple linear regression model inverse results. (**a**) Residual stress in the x-direction and (**b**) case depth.

When fitting x24 (as in Table 6) using a primary function of residual stress, case depth, and a cubic function of hardness, the coefficient of determination was greater than 0.95 with the highest accuracy. The weight of the influence of residual stress, hardness, and case depth on the magnetic signal decreased in order. The results of the residual stresses from the inverse of the selected x24 equation are shown in Figure 15a, with the R2 greater than 0.95 and the RMSE less than 7 MPa, and the inverse results are better than those predicted by the MLR model. The inverse case depth results are shown in Figure 15b, where the case depth is less than 0.15 mm and the inverse results are more accurate than the MLR model. The RMSE is less than 0.06 mm. It can be used to complement the MLR model.

## 5. Conclusions

Bi-directional magnetic parameters (extracted from TMF, MBN, and IP signals) and the profiles of target properties (the bi-directional residual stress, surface hardness, and case depth) in hardened steel sheets were experimentally obtained. Three methods, Linear Curve Fitting, MLR, and Single Parameter Fitting Inverse, were investigated for the quantitative assessment of the target properties.

Pearson correlation analysis was carried out to find those parameters that show an approximately linear dependence on the target properties. Correlation analysis in the y-direction shows that when influenced by residual stress alone, the correlation coefficients of all magnetic parameters with residual stress are greater than 0.8, except for x1, x14, x17, and x20, indicating that the three magnetic signals are sensitive to residual stress. Correlation analyses in the x-direction were influenced by a combination of target properties. Typically, the MBN and IP signals were influenced by both microstructure and residual stress. The x16 of MBN and x20–x25 of IP signals were closely related to hardness and residual stress. In addition, x15 of MBN has a strong correlation with all three target properties. In contrast, x2, x7, x8, and x9 of the TMF signal were closely correlated with hardness and case depth, indicating that the TMF signal is more sensitive to microstructural changes.

Prediction of the target properties was achieved by constructing the MLR model using magnetic parameters that were highly correlated with them. Residual stresses in the y-direction were predicted with higher accuracy (R^2^ = 0.95243) than in the x-direction (R^2^ = 0.89433), and the RMSE of the residual stresses in both directions was less than 11 MPa. The hardness prediction was also accurate (R^2^ ≈ 0.95, RMSE = 7.7939 HV0.2). However, the case depth was not predicted accurately enough at smaller depths. To improve the prediction of the x-direction stress and case depth, the regression model based on single magnetic parameters was developed for inverse calculation. The R^2^ of the x-direction residual stress increased to 0.95857, and the RMSE was reduced to 6.4512 MPa. The absolute error (AE) for case depths < 0.15 mm was <0.04 mm, providing a complementary approach to MLR predictions.

The proposed multifunctional magnetic testing technique provides an efficient and non-destructive approach to the quantitative prediction of residual stress, surface hardness, and case depth in medium carbon steel plates. This prediction method is particularly suitable for mechanical components requiring high surface hardness and wear resistance, such as shafts, gears, molds, and guide rails. For workpieces with relatively uniform geometry, moderate curvature variations, and sufficient thickness (exceeding the magnetic skin depth), the technique can directly predict the surface hardness and residual stress distributions while evaluating case depth uniformity. However, for components with complex geometries (e.g., significant curvature variations or large thickness differences), additional considerations are required to account for the influence of geometric factors on the magnetic signals. Local calibration modeling may be required to improve the measurement accuracy. Future research should focus on optimizing the magnetic testing technique for complex-shaped workpieces and exploring its applicability in surface hardness detection to expand its engineering applications.

## Figures and Tables

**Table 1:** Chemical composition analysis of tested specimens.

Material	C	Mn	Si	S	P	Cr	Mo	Ni	V	Cu
45	0.49	0.50	0.17	0.0012	0.014	0.020	0.0010	0.0052	<0.001	<0.01

**Table 2:** Measurement results of residual stress, surface hardness profiles, and effective case depth.

Point Number	1	2	3	4	5	6	7	8	9	10	11
Residual stress in the x-direction (MPa)	−607	−619	−648	−669	−673	−710	−666	−642	−633	−624	−597
Residual stress in the y-direction (MPa)	−851	−874	−890	−937	−977	−988	−954	−925	−900	−884	−863
Surface hardness (HV0.2)	622	704	669	697	677	675	671	697	705	680	589
Effective case depth (mm)	0.07	0.26	0.10	0.27	0.28	0.16	0.24	0.17	0.28	0.29	0.20

**Table 3:** Feature parameters extracted from TMF, MBN, and IP signals.

Signal Types	Indicators	Magnetic Parameters	Descriptions	Unit
Tangential magnetic field signal	x1, x2, x3, x4	A1, A3, A5, A7	Amplitudes of the 1st, 3rd, 5th, and 7th harmonics	A/cm
	x5, x6, x7	P3, P5, P7	Phases of the 3rd, 5th, and 7th harmonics	rad
	x8	UHS	Sum of the amplitudes of the 3rd, 5th, and 7th harmonics	A/cm
	x9	K	Distortion factor	%
	x10	Hro	Harmonic amplitude at the tangential magnetic field crossing the zero point	A/cm
	x11	Hco	Amplitude of the harmonic component of tangential magnetic field at the first zero-crossing point	A/cm
Barkhausen noise signals	x12	Mmax	Peak height of the MBN butterfly curve	V
	x13	Hcm	Peak position of the MBN butterfly curve	A/cm
	x14, x15, x16	DH75M, DH50M, DH25M	Full width at 75%, half, and 25% of maxima of MBN butterfly curve	A/cm
	x17	Mr	Intercept of MBN envelope at the vertical axis	V
	x18	Mmean	Mean value of MBN envelope for a single magnetization cycle	V
Incremental magnetic permeability signal	x19	μmax	Peak height of the IP butterfly curve	V
	x20	Hcμ	Peak position of the IP butterfly curve	A/cm
	x21, x22, x23	DH75μ, DH50μ, DH25μ	Full width at 75%, half, and 25% of maxima of IP butterfly curve	A/cm
	x24	μr	Intercept of IP envelope at the vertical axis	V
	x25	μmean	Mean value of IP envelope for a single magnetization cycle	V

**Table 4:** Coefficients of multiple linear regression model.

Indicators	Model Coefficients			
	Model xRs	Model yRs	Model SH	Model ECD
constant	−6.95 × 10^3^	1.80 × 10^4^	−9.93 × 10^4^	2.58
x1	0	0	6.41 × 10^3^	0
x2	0	3.34 × 10^4^	−1.71 × 10^5^	11.96
x3	0	1.43 × 10^4^	0	0
x6	−94.89	−5.66 × 10^2^	0	0
x7	0	0	1.33 × 10^3^	0
x8	0	0	0	−17.10
x9	0	−5.92 × 10^5^	2.90 × 10^6^	0
x10	0	3.56 × 10^3^	0	0
x12	1.19 × 10^4^	0	0	0
x15	0	29.65	0	0.1957
x16	0	−15.44	0	0
x17	9.33 × 10^3^	0	0	0
x18	−1.46 × 10^5^	2.03 × 10^4^	0	0
x20	4.66 × 10^2^	0	0	0
x21	0	−4.86 × 10^3^	−1.54 × 10^3^	0
x22	0	2.71 × 10^3^	8.49 × 10^2^	0
x23	0	−4.62 × 10^2^	−2.68 × 10^2^	0
x24	5.27 × 10^4^	0	0	0
x25	0	2.01 × 10^5^	0	0

**Table 5:** Multiple quadratic fitting results of magnetic feature parameters.

Indicators	Parameters	Primary Coefficient			Quadratic Coefficient			Constant	R^2^
		$x_{ECD}$	$x_{SH}$	$x_{xRS}$	$x_{ECD}^{2}$	$x_{SH}^{2}$	$x_{xRS}^{2}$		
x2	A3	−2.43 × 10^−2^	1.02 × 10^−3^	0	0	−8.30 × 10^−7^	0	0.22	0.7132
x9	K	−1.84 × 10^−3^	0	0	0	−3.52 × 10^−9^	0	3.29 × 10^−2^	0.7534
x21	DH75μ	2.2709	0.0337	−0.0261	−4.7213	0	0	−5.8437	0.8890
x22	DH50μ	4.6061	0.0655	−0.0541	−9.8316	0	0	−15.2836	0.8607
x23	DH25μ	0	−0.0418	−0.1106	0	0	−0.0001	2.7874	0.9272
x24	μr	0	2.07 × 10^−4^	−2.09 × 10^−5^	0	−1.54 × 10^−7^	0	−1.13 × 10^−2^	0.9247
x25	μmean	0	4.09 × 10^−5^	−2.20 × 10^−6^	0	−3.06 × 10^−8^	0	1.59 × 10^−2^	0.8811

**Table 6:** Multiple cubic fitting results of magnetic feature parameters.

Parameters	Primary Coefficient			Quadratic Coefficient			Cubic Coefficient			Constant	R^2^
	$x_{ECD}$	$x_{SH}$	$x_{xRS}$	$x_{ECD}^{2}$	$x_{SH}^{2}$	$x_{xRS}^{2}$	$x_{ECD}^{3}$	$x_{SH}^{3}$	$x_{xRS}^{3}$		
μr	2.84 × 10^−3^	8.89 × 10^−3^	−2.51 × 10^−5^	0	−1.35 × 10^−5^	0	0	6.82 × 10^−9^	0	1.89	0.9555

## Data Availability

The data presented in this study are available upon request from the corresponding author.
